# A cluster-randomized trial of interventions for adolescent mental disorders in Zimbabwe

**DOI:** 10.1186/s12888-025-06755-x

**Published:** 2025-07-02

**Authors:** Rhulani Beji-Chauke, Victoria Simms, Melanie Abas, Kelly Muzariri, Webster Mavhu, Collin Mangenah, Ruth Verhey, Ephraim Chiriseri, Jermaine M. Dambi, Ricardo Araya, Helen A. Weiss, Frances M. Cowan, Dixon Chibanda

**Affiliations:** 1 Friendship Bench Zimbabwe, 4 Weale Rd, Milton Park, Harare, Email Zimbabwe; 2 MRC International Statistics and Epidemiology Group, London School of Hygiene and Tropical Medicine, Keppel Street, London, WC1E 7HT UK; 3 Institute of Psychiatry, Psychology and Neuroscience, King’s College London, London, SE5 8AF UK; 4 Centre for Sexual Health and HIV/AIDS Research (CeSHHAR) Zimbabwe, 4 Bath Road, Belgravia, Harare, Zimbabwe; 5 Department of International Public Health, Liverpool School of Tropical Medicine, Liverpool, L3 5QA UK; 6 Department of Psychiatry, University of Zimbabwe College of Health Sciences, Parirenyatwa Grounds, Harare, Zimbabwe

**Keywords:** Adolescent, Common mental disorders, Clinical trial, Problem-solving therapy, Task-shifting, Depression, Anxiety, Global health, Low- and middle-income countries, Economic costs

## Abstract

**Purpose:**

Young people have low uptake of mental health. We compared two task-shifted mental health care models, i.e., adult Friendship Bench (FB) delivered by community health workers and Youth Friendship Bench (YouFB) delivered by trained university students in Harare, Zimbabwe. We hypothesised that the peer-delivered YouFB would have greater uptake and effectiveness in managing common mental disorders (CMDs) in 16–19-year-olds compared to the standard FB model. We also aimed to evaluate the reach, fidelity, acceptability and cost of the YouFB compared to standard FB.

**Methods:**

We conducted an open-label cluster-randomised, hybrid type-2 implementation trial with cost analysis in 26 primary care clinics and their surrounding communities. Facilities were randomised 1:1 to FB or YouFB. The primary implementation outcome was uptake, defined as the proportion of adolescents aged 16–19 offered FB sessions for treatment of CMD who completed at least one FB session. Secondary implementation outcomes included reach, fidelity, and acceptability. The main clinical outcome was the clinical effectiveness of YouFB vs. FB at six months, assessed by changes in Shona Symptom Questionnaire (SSQ-14) scores. We also carried out a cost analysis from a societal perspective. Acceptability was evaluated qualitatively using in-depth interviews. Reach was calculated as the number of adolescents receiving FB sessions per clinic day.

**Results:**

Uptake in the FB and YouFB arms was 86.6% (187/216) and 95.6% (220/230), respectively (primary outcome). The number of completed FB sessions (feasibility) was higher in the YouFB arm than the FB arm (cluster-level mean prevalence 96.7% vs. 85.8%, prevalence ratio = 1.13; 95% CI:0.98–1.30). Among 528 trial participants, adjusting for baseline score, gender, education, marital status, employment and HIV status, the proportion of participants with SSQ-14 score ≥ 8 was similar by arm after six months, adjusted odds ratio = 0.65 (95% CI: 0.36–1.17). Total program costs were higher in the YouFB arm. Process evaluation found the YouFB to be highly acceptable.

**Conclusions:**

A youth-focused Friendship Bench intervention is feasible and acceptable. Recipients highly valued the ability to connect with a same-age peer and its easy accessibility. However, further intervention optimisation is needed to improve its clinical and cost-effectiveness.

**Trial registration:**

This trial was prospectively registered on 21/08/2018 with the Pan African Clinical Trial Registry database. Registration no PACTR201808181810124.

**Supplementary Information:**

The online version contains supplementary material available at 10.1186/s12888-025-06755-x.

## Background

Common mental disorders (CMDs), namely anxiety and depression, are the leading cause of disability-adjusted life years globally [1]. CMDs often first present in mid-to-late adolescence, and if well managed, long-term sequelae can be mitigated [2]. In a systematic review, prevalence based on 37 studies involving sub-Saharan adolescents was estimated at 26.9% for depression and 29.8% for anxiety [1]. CMDs, if untreated, may result in school dropout, substance use, and sexual risk-taking, all of which in sub-Saharan Africa, put young people at increased risk of HIV acquisition [3, 4]. Additionally, adolescents with CMDs are less likely to seek help compared to other age groups, and treatment dropout rates are high [5].

Mental health service provision in many low- and middle-income countries (LMICs) is inadequate, and specialist youth-friendly mental health care is unavailable [6]. This is compounded by the absence of evidence-based, cost-effective, scalable and easily accessible youth-friendly interventions [7]. Attempts to improve adolescents’ mental health in LMICs have been impeded by a lack of policies as well as funding for specialist resources and a lack of contextualised evidence regarding culturally appropriate psychosocial interventions [8]. Also, few evidence-based mental health programs have been scaled up with success globally [9].

The Friendship Bench (FB) is an evidence-based, low-threshold psychological intervention delivered to individuals by community health workers (CHWs) [10]. It provides problem-solving therapy and behavioural activation within a community setting and primary healthcare facilities [10]. Specifically, the Friendship Bench intervention focuses on exploring and understanding the clients’ situational context through talk therapy, positive relational experience through being listened to non-judgementally, and intrapersonal growth towards strength and ability through goal-oriented learning [11]. The FB was developed in Zimbabwe to bridge the treatment gap for CMDs and is highly effective in adults [10]. In a randomised controlled trial among 573 adults, FB reduced CMDs at six months post-randomisation compared to standard care (adjusted risk ratio [ARR] 0.21; 95% CI 0.15–0.29; *p* < 0.001) [10]. It was equally effective in participants aged 18–22 as in older adults [10]. However, a low proportion of participants were aged 18–22. Therefore, increasing uptake and further assessing the effectiveness of the intervention in adolescents and young adults is paramount.

To tackle the rise of CMDs in youth, the Friendship Bench developed the Youth FB (YouFB) intervention. The YouFB intervention was developed using a theory of change workshop where young people and other stakeholders were engaged to provide input to the intervention [12]. The YouthFB is a bespoke, peer-delivered intervention that aligns with a recommendation based on a case series on youth engagement: to collaborate with young people early in the research process to allow for meaningful involvement [12]. In this paper, we present the results of a trial comparing the uptake, acceptability, feasibility, reach, effectiveness and economic costs of the original FB intervention (standard of care) compared with an adapted version of the FB delivered by youth lay health workers (YouFB) in Zimbabwe.

## Methods

### Trial design and setting

The study was a pragmatic cluster-randomised controlled trial (CRT) in Harare, Zimbabwe. In this trial, a cluster was defined as the geographic area within the proximity of a primary healthcare facility. This encompassed the adjacent communities, including schools, church halls and community centres frequented by young individuals.

### Participants

Clients were eligible to participate if they were aged 16–19 and scored ≥ 8/14 (8 or above) on the SSQ-14, a locally developed and validated screening tool for CMDs [13]. Potential participants were excluded if they could not comprehend the study in either English or Shona (local language), were currently in psychiatric care, were presenting with suicidal intent or psychosis, showed signs of intoxication or had end-stage AIDS. Those excluded for medical reasons were referred appropriately. Eligible participants were asked to provide written informed assent (if < 18 years) with their primary caregiver also providing written consent. Those aged 18–19 years were asked to provide written consent.

### Trial intervention

The FB and YouFB programs are both based on the original clinical trial, which emphasised a practical approach to problem-solving therapy where participants are taught a structured approach to identifying problems and finding a workable solution [10]. The intervention consists of 4–6 sessions of problem-solving therapy delivered weekly, and is based on indigenous concepts: *kuvhura pfungwa*,* kusimudzira*, and *kusimbisa* [14, 15]. *Kuvhura pfungwa* which translates to ‘opening the mind’ is the initial step which ‘opens the client’s mind’ to be aware of the problems they are facing [14, 15]. This is followed by *kusimudzira* (uplifting) where the client is guided by the YouFB buddie or adult CHW to select one problem to work on and then go through a process of brainstorming and selecting a solution [14, 15]. The final concept, *kusimbisa* (strengthening), empowers the client to come up with a detailed plan on how to implement the solution [14, 15]. A detailed description of the intervention is provided in supplementary Table 4. The difference between the FB and YouFB was in the delivering agent and setting as follows:

(i) **FB** - The Friendship Bench was conducted by existing adult CHWs experienced in FB delivery in the 13 standard-of-care arm clinics. Adult CHWs received additional training around parental consent, reporting cases of abuse, confidentiality and dealing with minors in distress on providing the FB intervention to adolescents, ensuring they were aware of their specific needs.
(ii) **YouFB** - In the 13 communities offering the adapted YouFB intervention, services were provided by trained, mixed-sex YouFB “buddies”. These were undergraduate psychology students serving a 10-month attachment with the Friendship Bench. They were selected following a competitive interview that assessed communication skills and a basic understanding of psychoeducation. The role of buddies was to raise mental health awareness within communities, screen young persons at risk of CMD, and provide FB sessions in clinical (primary healthcare facilities) and non-clinical settings (e.g., churches, schools (after hours) and community centres). Young people at risk of CMD were enrolled into the YouFB intervention.

All 16–19-year-olds presenting to the FB clinics (standard of care arm) or YouFB providers (intervention arm) were screened for CMD, and those at risk (scoring *≥* 8 on the SSQ) were invited to participate in the study. Intervention providers in both arms were supervised and supported by more experienced staff who were contacted by mobile phone if clients presented with “red flags” such as scores ≥ 11 on the SSQ-14, especially focusing on self-reports of suicidality or hallucinations. Those providing supervision included FB trainers, adult CHWs who were not delivering the intervention in this trial and clinicians.

The original plan was for all clusters to provide services for six months, but this did not happen due to reasons described later in this paper. From June to November 2019, YouFB buddies rotated among intervention clinic catchment areas every two weeks. Meanwhile, screening and uptake of usual FB services by adolescents were measured in one (1) FB clinic at a time for two weeks per clinic. In November 2019, the service was scaled up to 12 communities/clinics simultaneously (6 YouFB communities and 6 FB clinics), and in January 2020, an additional five (5) communities/clinics (3 YouFB communities, 2 FB clinics) were added. Enrolment closed on 27 February 2020.

### Outcomes

This was a hybrid type-2 implementation trial, as it had a dual focus on implementation and clinical effectiveness [16]. The primary implementation outcome was uptake, defined as the proportion of adolescents aged 16–19 offered FB sessions for treatment of CMD who completed at least one FB session. Secondary implementation outcomes included feasibility, reach, fidelity, and acceptability. Feasibility was defined as the number of completed sessions per arm. Reach was defined as the number of adolescents given FB sessions per cluster-day. Fidelity was operationalised as the extent to which all study and intervention components were implemented on schedule and as planned. Last, acceptability was defined as the extent to which counsellors and adolescents perceived and valued the intervention (YouFB). The primary clinical outcome was assessed as a difference in endline SSQ-14 score between arms, as both a binary outcome (proportion of participants with SSQ-14 score ≥ 8) and a continuous outcome (mean SSQ-14 score). Secondary clinical outcomes were similarly defined as differences in depression, anxiety, and disability as proportions with scores above cut-off points on the Patient Health Questionnaire-9 (PHQ-9), Generalized Anxiety Disorder-7 (GAD-7) and WHO Disability Assessment Schedule (WHODAS), respectively (Supplementary Table 2). We also evaluated the cost of the two interventions by estimating resource use data. Cost estimation was done from a societal perspective, which includes patient costs (direct and indirect costs collected at clinic attendance), the cost of the LHWs, and other provider costs.

### Sample size

We planned to measure the proportion of all clients who were aged 16–19 years by group to assess the impact of offering YouFB on the age distribution of clients. A sample size of 30 clusters would have provided 92% power to detect a difference in the number of clients seen per cluster of 20 in the FB arm versus 30 in the YouFB arm, assuming a standard deviation of 15 and a between-cluster coefficient of variation of 0.25. The planned sample size for the effectiveness trial was 375 participants in the YouFB arm (median 23 per cluster) and 125 in the control (FB) arm (median 10 per cluster). This planned sample size of 500 participants would have provided a 95% CI of 3.5–4.1 around a mean SSQ score of 3.8 at six months (SD = 3.6) as seen in the original FB trial [10] and provided 80% power to detect a difference of 1.3 points in mean SSQ (effect size = 0.36) between the FB and YouFB strategies, assuming an intra-cluster correlation of 0.05.

### Randomisation

The randomisation process included initially listing all eligible clusters based on a list provided by the Harare City Health Department. This was followed by blindly drawing one of two labelled (A & B) coloured balls from a black bag to assign the clusters to either the (A) control or (B) intervention arms. Twenty-six clusters were randomised 1:1 to provide either usual FB care or YouFB using minimisation to achieve balance on population size, clinic catchment size, HIV prevalence, and the number of 16-19-year-olds attending the FB during the adult scale-up implementation across Harare. The randomisation of an initial 24 Harare clusters was done on 10 August 2018 at the University of Zimbabwe-Research Support Centre in the presence of the senior District Health Promotion Officer (DHPO), adult CHWs, DHPOs and FB team members. The intention was to randomise a further 6 clusters in the cities of Chitungwiza (located on outskirts of Harare) and Gweru (∼300 km from Harare) for a total of 30. However, this was not possible due to delays in obtaining ethical approval. Instead, two additional Harare clusters were randomised on 10 May 2019, bringing the total to 26. This was an open-label trial. Allocation was not concealed from participants or staff, and the analysis was unblinded due to the nature of the data.

### Procedures

Trained research assistants screened, enrolled, and obtained consent from participants following standardised operation procedures. Enrolment was from 8 June 2019 to 27 February 2020 using the community wards as sampling frames. Depression was measured using the PHQ-9, a scale that has been widely used in Africa and validated for Zimbabwe with a cut point of 11 and above [13]. Participants who scored ≥ 8 on the SSQ-14 had the PHQ-9 administered to determine the presence of depression and its severity. Participants with severe depression symptoms (scoring ≥ 11 on the SSQ-14 and/or responding “Yes” to suicidal thoughts) were referred to the next level of care, including possible referral to a clinical psychologist and/or psychiatrist. Additionally, the GAD-7 [17] and WHO-DAS [18], cut point 20/100 were used. The GAD-7, an instrument to assess anxiety severity, has been validated in Zimbabwe and found to have good psychometric properties with a cut point of 10 and above [13]. In the nested effectiveness trial, the outcomes were SSQ-14, PHQ-9, GAD-7 and WHODAS scores six months after enrolment to the FB or YouFB. We audio-recorded each client’s first counselling session with a YouFB buddy, and the assessment was done by FB trainers using a pre-designed checklist to establish fidelity. A similar approach was successfully used previously [10].

### Data management

The screeners recorded on paper screening logs (i) the number of adolescents aged 16–19 years attending for assessment, (ii) the number who screened positive using the SSQ-14, and (iii) the number initiating YouFB. Outcome data for effectiveness among trial participants were collected by research assistants using a computerised self-administered questionnaire administered on a tablet using Open Data Kit or over the phone (Supplementary file S2).

### Data analysis

#### Implementation outcomes

##### Quantitative analysis

We applied a mixed methods approach to analyse the implementation outcomes. The primary outcome was the proportion of eligible adolescents offered counselling who accepted it (uptake). The proportion was used rather than an absolute number of adolescents who accepted counselling because the number of cluster-days for available data differed between arms. The geometric mean cluster-level prevalence of completed sessions was compared between arms as a prevalence ratio. For the reach outcome, the number of adolescents counselled per arm was divided by the number of cluster days per arm to calculate a rate ratio.

##### Qualitative analysis

We also conducted a process evaluation to explore fidelity and acceptability. We conducted in-depth interviews with a purposive sample of 13 young people who responded positively to the intervention as measured by a change in their SSQ-14 scores and 13 who responded poorly; participants were drawn from both experimental and control arms. We also conducted additional interviews with interventionists i.e., 12 YouFB buddies (*n* = 6 male; *n* = 6 female) and two focus group discussions (FGDs) with adult CHWs (all female).

#### Clinical outcomes

To analyse the effectiveness of YouFB compared with FB, the endpoints were SSQ-14 (primary outcome), PHQ-9, GAD-7, and WHO-DAS, each analysed as continuous and binary variables. For continuous outcomes, standardised mean differences (SMD, effect size) and 95% confidence intervals (CI) were estimated using mixed-effects linear regression, adjusting for cluster as a random effect, and the baseline measurement of the outcome and other variables associated with the trial arm at baseline or with missingness. For binary outcomes, adjusted odds ratios and CI were estimated using analogous mixed-effects logistic regression. To adjust for clustering, we calculated the *p*-value and confidence intervals from a t-distribution rather than a z-distribution, with degrees of freedom as the number of clusters at follow-up (24) minus the number of cluster level parameters [19]. As a sensitivity analysis, we used multiple imputations to impute missing outcome data with 10 imputations.

For qualitative data, four (4) research assistants (2 male, 2 female) were initially trained by a senior researcher (WM). The research assistants interviewed YouFB buddies and adolescents, and WM facilitated the focus group discussions with adult CHWs. The interview guides were developed specifically for this study (Supplementary file S3). All interviews and discussions were audio recorded, and transcription and translation were conducted by the research assistants. Iterative qualitative data collection, processing and analysis informed a grounded thematic analytical approach.

#### Cost analysis

We measured, characterised, and compared the full economic costs of the YouFB against the FB. Our costing approach took the provider perspective and followed international costing guidelines for implementation during the trial period (June to December 2019). Actual program financial expenditures were analysed (line by line), categorised by input type and then allocated to the respective arm and site. We estimated the total program and annual cost for both the YouFB (*n* = 13) and FB (*n* = 13) sites. Actual program financial expenditures were analysed (line by line) in a specifically designed Microsoft Excel spreadsheet, categorised by input type, and then allocated to the respective arm and cluster. This top-down costing approach has been shown to more fully account for inefficiencies, downtime, and wastage, and involves stepwise allocation of actual expenditures from the program office level to the respective site and cost centres [20–22].

Programme resources were classified into start-up (stakeholder sensitisation, training of trainers, etc.), capital (initial training of both youth and adult LHWs, program equipment, etc.) and recurrent costs, including personnel, stationery supplies, communication expenses, promotional materials, and maintenance (Supplementary Table 3). Personnel costs included program management and staff time, psychologist time, supervision provided by Friendship Bench trainers, and other support and allowances for youth and existing adult LHWs, clinicians and DHPOs. Personnel costs were allocated based on interviews with staff and timesheet data. All capital costs (start-up, initial training, and equipment) were annualised using the standard 3% discount rate (Supplementary Table 3).

## Results

### Participant flow

In the FB arm, screening logs were available for 175 cluster-days in 12 clinics (median 10.5 days per cluster, range 6–29). In the YouFB arm, screening logs were available for 137 cluster-days in 12 communities (median 11 days per cluster, range 1–24). No logs were retained at the remaining two clusters (one per arm).

**Table 1:** Demographic characteristics of participants in the uptake trial

		FB arm (175 cluster-days)				YouFB arm (137 cluster-days)			
		Screened, n (%)	SSQ ≥ 8, n (%)	Offered counselling	Accepted counselling, n (%)	Screened, n (%)	SSQ ≥ 8, n (%)	Offered counselling	Accepted counselling, n (%)
	*N*	*583*	*269*	*216*	*187*	*653*	*310*	*230*	*220*
Gender	Male	255 (43.8)	111 (41.4)	89 (41.4)	78 (41.9)	372 (57.1)	167 (54.2)	125 (54.4)	124 (56.4)
	Female	327 (56.2)	157 (58.6)	126 (58.6)	108 (58.1)	279 (42.9)	141 (45.8)	105 (45.7)	96 (45.6)
	Missing	1	1	1	1	2	2	0	
Age	16	152 (26.2)	53 (19.9)	38 (17.8)	29 (15.6)	169 (25.9)	68 (22.0)	39 (17.0)	38 (17.3)
	17	137 (23.6)	58 (21.7)	43 (21.1)	36 (19.4)	194 (29.8)	84 (27.2)	59 (25.7)	57 (25.9)
	18	124 (21.4)	72 (27.0)	57 (26.6)	53 (28.5)	157 (24.1)	81 (26.2)	67 (29.1)	64 (29.1)
	19	167 (28.8)	84 (31.5)	76 (35.5)	68 (36.6)	132 (20.2)	76 (24.6)	65 (28.3)	61 (27.7)
	Missing	3	2	2	1	1	1	0	

### Primary implementation outcomes

**Fig. 1:**
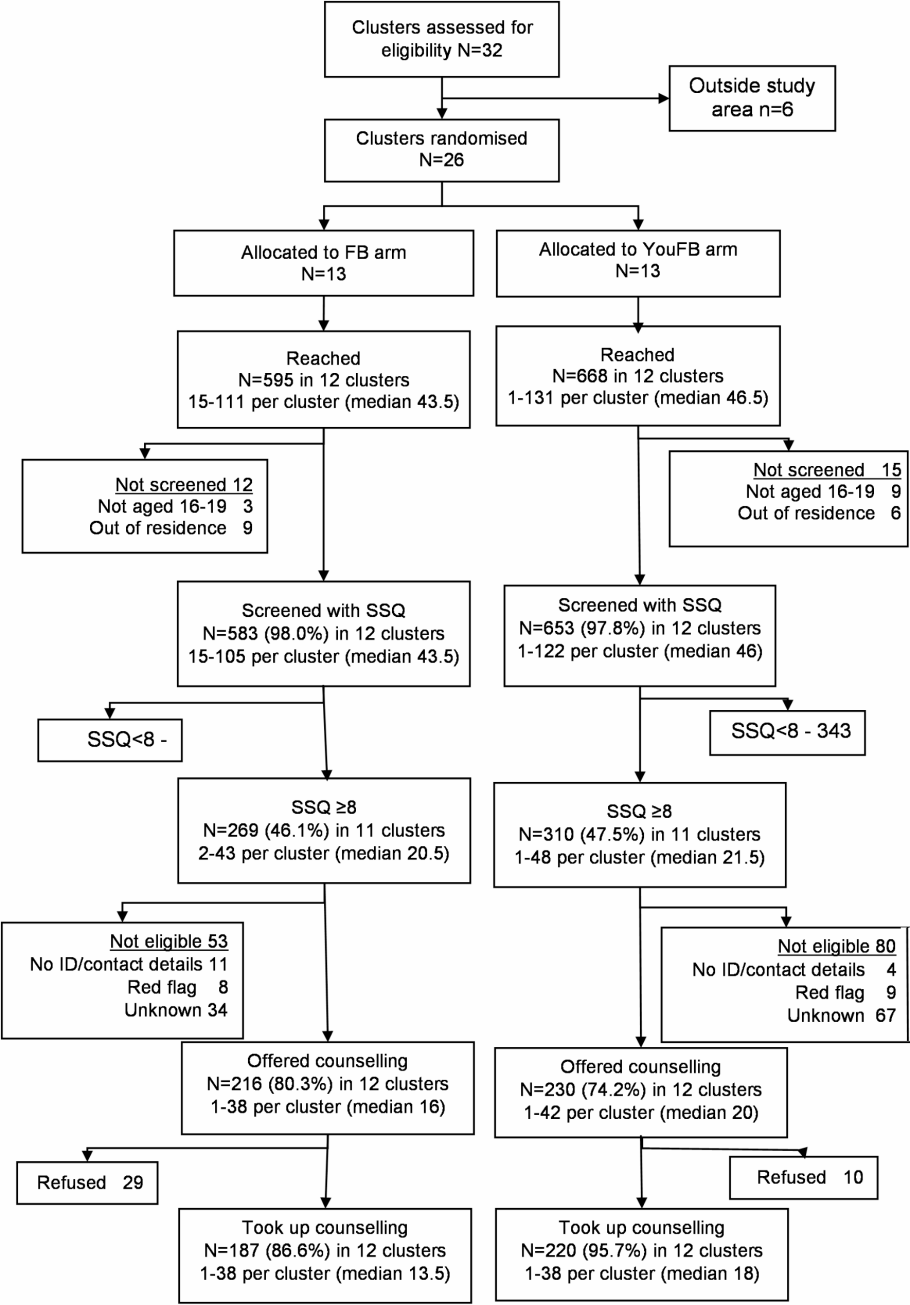
Flow chart of uptake trial

In the FB arm, 595 adolescents were contacted, of whom 583 were screened with the SSQ-14; of these, 269 had an SSQ score of ≥ 8, 216 were fully eligible and were offered counselling, and 187 accepted it and attended at least one session of FB intervention delivered by an adult lay counsellor (Fig. 1). In the YouFB arm, 668 adolescents were identified, 653 were screened with the SSQ-14, 310 had a score ≥ 8, 230 were fully eligible and were offered counselling, and 220 accepted it. In cluster-level analysis, the geometric mean percentage uptake was 96.7% in the YouFB arm compared to 85.8% in the FB arm (prevalence ratio 1.13, 95%CI 0.98–1.30, *p* = 0.10), as shown in Table 2.

**Table 2:** Effectiveness of the youfb on uptake of counselling among those eligible

Arm	Number who accepted counselling (Cluster-level mean % of those offered)	Prevalence ratio (95% CI)	*p*
FB	187/216 (85.8%)		
YouFB	220/230 (96.7%)	1.13 (0.98, 1.30)	0.10

The mean number of adolescents screened per day was 3 (IQR 2–6, range 0–28) in the YouFB arm and 3 (IQR 2–5, range 1–12) in the FB arm. In the FB arm, the recorded uptake of counselling was 187 adolescents in 175 cluster-days or 1.07 per cluster per day. In the YouFB arm, 220 adolescents received counselling in 137 cluster-days or 1.61 per cluster per day. The rate ratio was 1.50 (95%CI 1.23–1.84, *p* < 0.001, indicating higher reach in the YouFB arm. In the FB arm, most clients at every stage were female (56.2% of those screened, 58.6% of those eligible, 58.1% of those who accepted counselling) and in the YouFB arm, a lower proportion were female (42.9% of those screened, 45.8% of those eligible, 45.6% of those who accepted counselling (Table 1). Data on adherence (number of sessions attended) were not collated and recorded.

### Nested effectiveness trial recruitment and follow-up

**Fig. 2:**
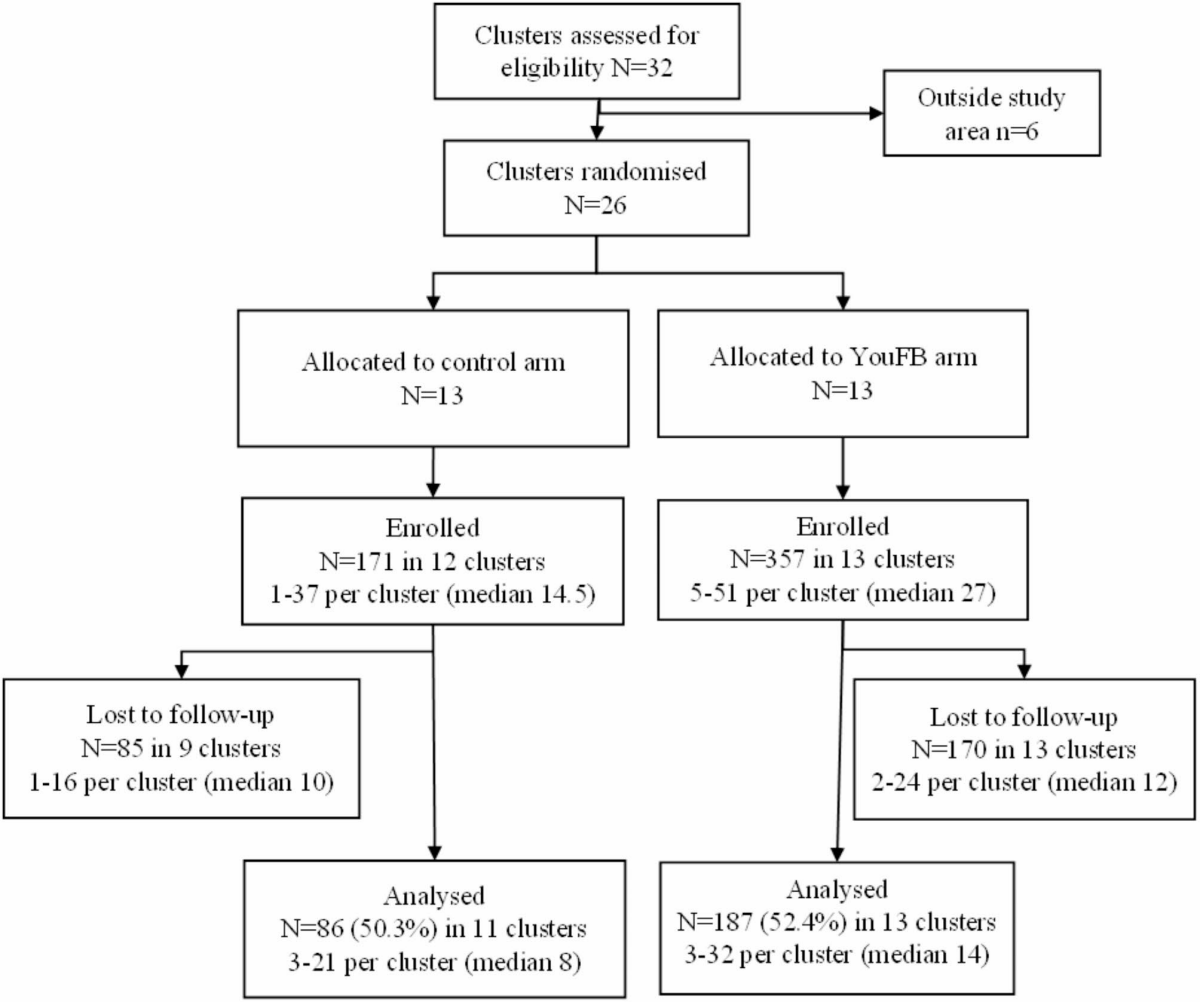
Flow chart of nested effectiveness trial

Of the 26 randomised clusters, *n* = 25 (96%) clusters enrolled participants into the nested effectiveness trial (from 1 to 51 participants per cluster), and total enrolment was 171 participants in the FB arm and 357 participants in the YouFB arm (See Fig. 2). Baseline characteristics of participants are shown in Table 3. The variables gender, education, marital status, employment status and HIV status showed some imbalance between arms or association with loss to follow-up and were adjusted for in subsequent analyses. The proportion of participants followed up was similar in the two arms (50.3% in the FB arm and 52.4% in the YouFB arm). This low proportion was due to the COVID-19 pandemic and a cholera outbreak described below. Follow-up data were collected from 6 November 2019 to 10 September 2020. The median length of follow-up was 144 days (IQR 113–210; range 34–420).

**Table 3:** Baseline characteristics of the nested effectiveness trial population by arm

		FB, *n* (%)	YouFB, *n* (%)
		171	357
SSQ-14	Mean	10.05	9.59
PHQ-9	Mean	8.66	8.76
	≥ 11	52 (30.4)	125 (35.0)
GAD-7	Mean	10.28	10.51
	≥ 10	83 (51.9)	203 (58.5)
WHODAS	Mean	0.30	0.33
	≥ 20	105 (65.6)	274 (79.0)
Gender	Male	63 (36.8)	197 (55.2)
	Female	108 (63.2)	160 (44.8)
Age	16	30 (17.5)	66 (18.5)
	17	30 (17.5)	74 (20.7)
	18	52 (30.4)	104 (29.1)
	19	59 (34.5)	113 (31.7)
Education	Grade 7 or below	16 (9.4)	24 (6.7)
	O level	141 (82.5)	261 (73.1)
	A level or above	11 (8.2)	72 (20.2)
Marital status	Single	138 (80.7)	334 (93.6)
	Married	26 (15.2)	14 (3.9)
	Divorced/ widowed	7 (4.1)	9 (2.5)
Employed	No	154 (90.1)	333 (93.3)
	Yes	17 (9.9)	24 (6.7)
HIV status	Positive	13 (7.6)	4 (1.1)
	Negative	68 (39.8)	131 (36.7)
	Unable to disclose	19 (11.1)	32 (9.0)
	Unknown	71 (41.5)	190 (53.2)
Drinks alcohol	No	156 (91.2)	317 (88.8)
	Yes	15 (8.8)	40 (11.2)

### Primary and secondary clinical outcomes

There was no evidence of a difference between arms in the proportion of participants with SSQ-14 score ≥ 8 at endline (aOR = 0.65, 95% CI 0.36–1.17; *p* = 0.13) or in mean SSQ-14 score at endline (AMD=-0.73, 95% CI -2.06-0.59; *p* = 0.25) (Table 4). Similarly, there was no evidence of a difference between arms in the proportion of participants with the other outcomes. The multiple imputation sensitivity analysis results were like the main analysis (Supplementary Table 1). The variables used for imputation were the baseline measures of the continuous outcome variables (SSQ-14, PHQ-9, GAD-7, and WHO-DAS), plus sex, age, HIV status, marital status, education, and employment status.

**Table 4:** Impact of intervention arm on outcomes in the nested effectiveness trial

Outcome	YouFB arm (*n* = 187)	FB arm (*n* = 86)	Unadjusted mean difference or odds ratio (95% CI)	*p*-value	Adjusted mean difference or odds ratio (95% CI) ^a^	*p*-value
**Primary outcomes**						
Proportion with SSQ-14 score ≥ 8 at endline	35.8% (67/187)	47.7% (41/86)	0.61 (0.35, 1.06)	0.06	0.65 (0.36, 1.17)	0.13
Mean SSQ-14 score at endline (SD)	6.02 (3.34)	6.90 (3.62)	-0.94 (-2.34, 0.49)	0.18	-0.73 (-2.06, 0.59)	0.25
**Secondary outcomes**						
Proportion with PHQ-9 score ≥ 11 at endline	16.4% (11/67)	29.3% (12/41)	0.46 (0.10, 2.12)	0.29	0.68 (0.13, 3.61)	0.63
Mean PHQ-9 score at endline (SD)	7.48 (3.69)	9.12 (4.67)	-1.42 (-4.06, 1.22)	0.27	-1.06 (-3.90, 1.77)	0.44
Proportion with GAD-7 score ≥ 10 at endline	35.3% (66/187)	32.6% (28/86)	1.13 (0.64, 2.00)	0.66	1.19 (0.63, 2.26)	0.57
Mean GAD-9 score at endline (SD)	7.74 (4.49)	7.64 (4.66)	-0.17 (-1.69, 1.34)	0.81	0.11 (-1.19, 1.41)	0.86
Proportion with WHO-DAS score ≥ 20 at endline	55.6% (104/187)	52.3% (45/86)	1.03 (0.51, 2.08)	0.93	1.02 (0.49, 2.10)	0.96
Mean WHO-DAS score at endline (SD)	0.25 (0.16)	0.25 (0.16)	-0.01 (-0.07, 0.05)	0.63	-0.02 (-0.09, 0.05)	0.58

^a^ adjusted for baseline value, gender, education, marital status, employment status and HIV status

### Secondary implementation outcomes

#### Acceptability of intervention providers

Discussions with adolescents in the YouFB arm showed overwhelming acceptance of YouFB buddies. Adolescents considered the YouFB buddies as peers; they had a better appreciation of the problems they raised, which might otherwise be considered “trivial” by adult CHWs. As one participant put it, *‘The issues that young people face might seem not so important to the “grannies” but these can be best understood by YouFB buddies who may have experienced the same problems recently and found some solutions…’* (18-year-old male). Overall, YouFB buddies were perceived to be more empathetic to adolescents than adult CHWs, and beneficiaries enjoyed the rare experience of being listened to (with empathy).

Adult CHWs were perceived as judgmental by adolescents. Adolescents felt that if counselled by adult CHWs, they would need to suppress issues that are considered socially unacceptable by adults, e.g., relationship, drug, and sexual-related issues. *‘…For example*,* relationship problems. She will say*,* “It is not the right time for you to be in a relationship"’* (16-year-old female). Adult CHWs acknowledged that with certain issues, adolescents likely opened up more to YouFB buddies. *‘…Issues related to “meeting on the sleeping mat” [having sex]… When an adolescent is talking to you about these issues*,* they do mention them*,* but for them to “go deeper”*,* they won’t. So*,* I think if we “refer” them to younger counsellors*,* I think they will be able to explain in greater detail because they will be almost the same age’* (adult CHW, FGD 01). That the age of the provider mattered was seen as critical by all groups of respondents.

Male adolescents particularly appreciated the opportunity to discuss their problems with male peers. They felt that there were specific problems they would not feel comfortable discussing with any adults or with female providers (e.g., sexual-related). Of note, adult female CHWs noted that they had difficulties handling sexual-related issues raised by males and wished they could have been paired with male counterparts.

#### Perceived YouFB program impact

Given the stigma associated with both clinic visits and a mental health session, adolescents liked having sessions within a community setting, as the meeting place was inconspicuous. They described how the YouFB program affected several aspects of their lives. Some described how they became better able to solve their long-standing relational problems. *‘I had a sour relationship with my father. After the sessions*,* I was able to approach my father and have a discussion with him…I never expected that one day I would reconcile with my father’* (17-year-old male). Another adolescent mentioned how the sessions and solutions derived from them had helped in overcoming drug use. Some solutions facilitated entrepreneurship. *‘Following the solution I adopted*,* my aunt is now “hoarding” [buying] me clothes from Zambia which I resell*,* and I am now able to make money’* (19-year-old female). Adolescents particularly enjoyed being encouraged to autonomously decide and act on their solutions, a deviation from the norm where adults often decide for them.

YouFB buddies appreciated the opportunity to work with young people and explained that the experience had equipped them with life skills to deal with their problems. ‘*Whilst providing services*,* I have also been empowered because*,* as a counsellor*,* it does not mean l also don’t go through problems or do not get depressed. Simply because l know this “PST” (problem-solving therapy)*,* I will be able to help myself and come out of any situation…”* (YouFB buddy, #1). Another stated, *‘PST can be used at an individual level*,* and one can administer PST to themselves and come up with solutions to the problems at hand’* (YouFB buddy, #12). Nearly all YouFB buddies highlighted their readiness to handle any future challenges.

As YouFB buddies were undergraduate psychology students on attachment, they stated this role helped them apply and appreciate theoretical issues they had learnt in their studies. *‘Through being a YouFB buddy*,* I got the opportunity to get a practical appreciation of social and psychological issues as well as actually practice what we were taught at college’* (YouFB buddy, #6). They also noted that the role enabled them to undertake relevant work that would likely influence their future career aspirations. *‘The work that we were doing helped me appreciate clinical psychology more*,* and I am now more determined than before to become a clinical psychologist’* (YouFB buddy, #9).

Some YouFB buddies described how their own resilience had been enhanced by listening to adolescents’ problems. *‘The program made me feel more confident in life because if I compare my problems with those of other young people*,* I realise mine are not that huge…’* (YouFB buddy, #11). Of course, the experience was sometimes depressing. *‘When doing PST sessions*,* there are stories which you hear which are so disturbing; for example*,* one client told me how she was denied food at home’* (YouFB buddy, #5). Finally, YouFB buddies mentioned that they felt less able than adult CHWs to assist with some problems (e.g., those bereavement-related), and participants to the YouFB agreed with this.

#### Implementation of the interventions and context

Implementation was hampered by various factors, including severe cholera and SARS-CoV-2 outbreaks, which resulted in lockdowns, restricted movements and service disruption. The interventions were not, therefore, delivered as intended. For example, the interventions were implemented over a few weeks in each cluster rather than over six months as originally planned. Additional challenges were structural. Several problems that young people brought up were linked to the wider socio-economic environment and, as expected, could not be addressed solely by the intervention. *‘We have what we call a “smart action plan” whereby we see this solution that one has come up with is measurable*,* is it attainable but then has some costs. What do we do now because this client is saying l have to travel to say Kadoma (~140km away) to tell my mum’* (YouFB buddy, #12).

In addition, some problems were embedded within young people’s relationships, with solutions to their problems often dependent on an adult relative. For example, when a young person’s action plan was visiting their long-deceased parent’s grave to enable closure, doing so depended on getting either permission and/or bus fare from their current caregiver, which rarely happened in practice.

### Economic costs

The total annual program cost of providing care to adolescents were $19,960 for standard FB and $47,163 for YouFB (Table 5). The operational costs were highly variable, and expenditure ranges per clinic were FB, $141 - $4,242 for FB, and $656 - $6,666 for YouFB. For YouFB, the major cost contributor was personnel, including psychologist counselling time (31%), program staff salaries (21%) and YouFB buddy allowances (17%), reflecting the additional time spent by program staff supporting the youth buddies as well as their $50 allowance per month.

**Table 5:** Total program costs

Input type	Standard FB cost	%	YouFB cost	%
**Start-up costs**				
*Stakeholder sensitisation*	$28	< 1%	$42	< 1%
*Training - ToT’s*	$33	< 1%	$125	< 1%
*Training - Adult LHW’s*	$51	< 1%	$0	< 1%
*Training - Youth LHW’s*	$0	< 1%	$1,070	2%
**Capital costs**				
*Building & storage*	$279	1%	$446	1%
*Equipment - Prog*	$1,056	5%	$2,074	4%
**Recurrent costs**				
*Personnel - HQ*	$2,184	11%	$2,184	5%
*Personnel - Intervention staff*	$11,930	60%	$11,930	25%
*Personnel - Psychologist time*	$0	< 1%	$7,799	17%
*Personnel - Adult LHW’s*	$1,357	7%	$0	< 1%
*Personnel - Youth LHW’s*	$0	< 1%	$14,676	31%
*Supplies - Prog stationery*	$446	2%	$930	2%
*Supplies - Communications*	$768	4%	$1,602	3%
*Supplies - Hats*,* bags*,* t-shirts*	$366	2%	$1,984	4%
*Vehicle operation & transport*	$719	4%	$681	1%
*Building operation*	$744	3%	$1,618	3%
**Total costs (recurrent)**	**$19**,**960**	**100%**	**$47**,**163**	**100%**

*Note that totals have been rounded off to the nearest US$

## Discussion

We conducted a cluster RCT to evaluate two models of delivering care for youth presenting with common mental disorders. Our trial showed a higher uptake of counselling among adolescents in the YouFB arm than in the FB arm; the difference was not statistically significant. There was no evidence of a difference in clinical effectiveness between the two groups. Implementing the youth-delivered intervention was relatively expensive. However, service delivery agents and recipients viewed the intervention as feasible and acceptable, and perceived it positively.

### Uptake

The non-difference in uptake between arms observed in this trial could be caused by the YouFB intervention not being implemented as intended. The intention was to deliver the YouFB intervention over six months in all locations. Because of clinic closures and other restrictions related to cholera and COVID-19 outbreaks, we implemented the trial over shorter periods in each location, which did not represent real-world implementation and likely affected uptake as young people had less opportunity to learn that the service was available due to lock-downs and a public health focus on COVID-19 and the cholera outbreak.

Systematic and scoping reviews attest to the importance of robust engagement of youth to create demand for mental health services [23–25]. Despite the inroads made in the past decade regarding increasing mental health coverage, the mental health care gap in low-income countries is still high [24]. In addition to resource shortages, the care gap is also driven by low awareness/mental health literacy, which is inherently associated with low uptake of interventions [24, 25]. Mental healthcare is also stigmatised; consequently, community- and facility-level awareness campaigns are essential for creating demand and uptake of youth mental health interventions [23–25]. Given the unforeseen disruptions in service delivery, the sensitisation/community awareness activities could not be implemented as planned. However, the process evaluation (qualitative) data supported the intervention’s feasibility and acceptability, suggesting the unforeseen logistical challenges may have negatively impacted the intervention’s uptake [12].

### Clinical effectiveness

Although the clinical effectiveness of the youth-delivered intervention was higher than that of adult lay counsellors, the difference was not statistically significant. These findings are congruent with a mixed scoping review and exploratory meta-analysis by Krause et al. (2021) to distil the active ingredients of problem-solving therapy (PST) in managing young anxiety and depression [26]. The meta-analysis showed that compared to control interventions, PST was associated with better depression outcomes; however, the differences were not statistically significant [g = − 0.34 (95% CI:−0.92 − 0.23) [26]. Studies analysed in the meta-analysis were of high risk of bias and heterogeneity [I^2^ = 88.37%, p<. 001]. PST is highly effective in managing adult anxiety and depression, but the lack of adequately powered clinical trials limits our understanding of the utility of PST in managing youth mental problems [26]. As found in our study, youth may lack the autonomy to implement solutions generated through PST, especially in more hierarchical cultures. A similar trial among Indian adolescents found only a modest effect of PST in improving idiographic (unique individual traits /attributes) priority problems but not self-reported mental health problems [23]. The lack of effect highlights the need for optimisation of PST interventions for managing youth anxiety and depression [23, 26]. PST is a core element of the YouFB intervention. There may be a need to add other evidence-based active ingredients to achieve better clinical treatment response. It is equally essential to determine training and supervision needs for counsellors delivering FB for youth with CMDs and to learn from the life experience that the older lay counsellors may have. However, a balance must be made between optimisation and complexity, particularly in low-income countries where mental health services are hugely task-shifted.

### Feasibility and acceptability

The qualitative component provided valuable insights into the intervention’s feasibility, acceptability, and perceived impact. Interviewees described various benefits of mental health services provision within a community setting and through peers. Peers notably facilitated empathic problem discussion and “opening up”, a term describing the process experienced by clients of FB on specific issues without concerns of being judged. A separate study within the same setting identified similar themes [11]. Also, a previous review explored the utility of therapeutic alliance in managing youth anxiety and depression and demonstrated youth’s preference towards same-aged peer counsellors to enhance therapeutic bonding [27]. On the other hand, the perceived closeness between client and supporter and comparatively little experience of the FB buddies puts them at risk of being negatively affected by the material their clients discuss with them. Thus, youth supporters must operate in a safe environment and receive ongoing training, debriefing and supervision [28]. Collectively, these findings highlight the inherent value of providing good quality adolescent mental health services through peer counsellors, a proposition also supported by scoping reviews and meta-analyses [23–25, 27]. Qualitative research teased out data on how the YouFB program had affected several aspects of their lives, thus providing nuanced information that would complement the quantitative evaluation.

A recurring theme is that adolescents have minimal agency to solve their problems, specifically those relating to finances, and rely on others to help them [11, 29]. Our process evaluation suggested that adolescents mostly identified problems they neither had the capacity nor resources to solve. However, PST is most effective when the counsellor helps the client choose a problem that is both meaningful and within their power to change [9]. A higher proportion of clients who took up the YouFB offer were male than those choosing the FB program. Mental health programs in this and similar settings struggle to attract males [30, 31]. Men traditionally access healthcare less than women [32], our findings buttress previous calls to take services to where men are to enhance uptake [33]. Further, having mixed-sex YouFB buddies enabled young males to seek services from male peers. This finding is supported by another study conducted among youth attending the Friendship Bench [34]. Going forward, blending older and younger providers will be critical, as young people described being more comfortable discussing specific topics with peers. At the same time, adult CHWs were better placed to deal with some specific issues (e.g., grief). Importantly, it is essential to provide ongoing training, supervision and support as peer supporters/counsellors must be trained to empower young people to work on problems within their control.

Our study supports previous evidence that young people gain fulfilment from being mental health support providers [11, 28]. However, the role can be challenging and emotionally draining, particularly when faced with problems they do not have the resources or experience to handle [12, 27]. It will be critical to keep the mental health needs of service providers in mind [28]. Of note, the needs of young peer supporters have been laid out in the TRUST framework, comprising Training, Referral pathways, Understanding the remit of their role, Supervision, and recognition that Talking helps [29]. Notably, the peer counsellors in this study were undergraduate psychology students on attachment. They acknowledged that having the opportunity to be trained and work in this role was personally and professionally beneficial.

### Cost analysis

The cost analyses show that the YouFB intervention had higher program costs than FB; this is not unusual when costing new programs [20–22]. The higher YouFB interventional costs were due to the additional inputs required, such as training of a relatively higher number of YouFB buddies, higher incentives ($50 monthly allowance per active YouFB buddy) compared to $10 airtime for adult CHWs (who already received a stipend from Harare City; their primary employer), psychologist counselling time and additional supplies (hats, bags, t-shirts). The FB counsellors are employed by the government through the Harare City Health Services Department as full-time health promoters [35]. The Friendship Bench provides them airtime to facilitate patient follow-ups and data uploading, as the government covers their salaries, hence the discrepancies in allowances. There is a need to consider integrating YouFB activities within government programming to allow cost-sharing and sustainability. Also, training costs were higher for YouFB buddies because they were new to the program, while FB counsellors, who were already experienced, received only refresher training. Importantly, newly trained YouFB buddies required additional training and support to ensure intervention fidelity; this drives the program costs higher.

Across the two arms and sites, average costs were susceptible to personnel time and the number of young beneficiaries supported by youth counsellors, indicating opportunities for cost reductions. A previous threshold analysis demonstrated that at least 3,413 service users per year are required to ensure cost-effective delivery of the FB counsellors-delivered intervention [35]. Program-level implementation data shows that more than 100,000 youths have received services from the YouFB buddies since 2020. The increased reach shows the YouFB model is potentially cost-effective over time. Overall, future health technology assessments are warranted to fully understand the health economic evaluation of the YouFB intervention as it is scaled up.

### Strengths and limitations

Our trial is one of the first studies to evaluate two models of delivering care for youth with common mental disorders as delivered in a real-world and resource-constrained setting. However, as with other evaluations of programmatic interventions, our trial was characterised by challenges to implementation, including severe cholera and SARS-CoV-2 outbreaks and high loss to follow-up. A limitation was that some logs were not correctly stored and were lost, reducing the sample size. No information was available on adherence, or the number of sessions received, preventing dose-response analysis. The challenges experienced in this study are not unique to the interventions tested here but are common during evaluations of real-world interventions, and all care has to be applied to mitigate them [33, 36].

## Conclusion

In conclusion, our findings show that an intervention developed for delivery within a primary healthcare setting by older community healthcare workers can be adapted for delivery within a community setting by trained youth counsellors. This adaptation is highly acceptable and feasible to deliver. There should be continual efforts to optimise the YouFB intervention by adding other evidence-based active ingredients (e.g. psychoeducation and behaviour activation), and continual evaluation of the intervention’s effectiveness as it is implemented at scale.

## Electronic supplementary material

Below is the link to the electronic supplementary material.

Supplementary Material 1

Supplementary Material 2

Supplementary Material 3

Supplementary Material 4

## Data Availability

The datasets used and/or analysed during the current study are available from the corresponding author on reasonable request.

## Abbreviations

CHW: Community Health Worker
CMD: Common Mental Disorders
DHPO: District Health Promotion Officer
FB: Friendship Bench
GAD-7: Generalised Anxiety Disorder
LMIC: Low and Middle-Income Country
PHC: Primary Healthcare Clinic
PHQ-9: Patient Health Questionnaire
PST: Problem Solving Therapy
RCT: Randomized Controlled Trial
SSQ-14: Shona Symptom Questionnaire
WHO: World Health Organization
WHO-DAS: World Health Organization Disability Assessment Schedule
YouFB: Youth Friendship Bench
