# Supplementary material for: A cluster-randomized trial of interventions for adolescent mental disorders in Zimbabwe
Source: BMC Psychiatry. 2025 Jul 2;25:673. doi: 10.1186/s12888-025-06755-x (PMC12220635; doi:10.1186/s12888-025-06755-x)
Supplement: Supplementary file 1 — Supplementary Material 1 [file 12888_2025_6755_MOESM1_ESM.docx]

Supplementary Table 1: Primary and secondary outcomes with multiple imputation (sensitivity analysis)

| **Outcome** | **Adjusted mean difference or odds ratio (95% CI)** | **p-value** |
| --- | --- | --- |
| **Primary outcomes** |  |  |
| Proportion with SSQ-14 score ≥8 at endline | 0.79 (.50, 1.27) | 0.34 |
| Mean SSQ-14 score at endline (SD) | -0.42 (-1.20, 0.36) | 0.29 |
| **Secondary outcomes** |  |  |
| Proportion with PHQ-9 score ≥11 at endline | 0.84 (0.41, 1.75) | 0.64 |
| Mean PHQ-9 score at endline (SD) | -0.22 (-1.31, 0.88) | 0.69 |
| Proportion with GAD-7 score ≥10 at endline | 1.05 (0.61, 1.79) | 0.87 |
| Mean GAD-9 score at endline (SD) | -0.07 (-1.14, 1.00) | 0.90 |
| Proportion with WHO-DAS score ≥20 at endline | 1.01 (0.59-1.72) | 0.97 |
| Mean WHO-DAS score at endline (SD) | -0.00 (-0.04, 0.03) | 0.81 |

Supplementary Table 2: Trial outcome definitions

| **Category** | **Classification** | **Operationalization/indicators** |
| --- | --- | --- |
| Implementation | Primary outcome | 1. Uptake - proportion of adolescents aged 16–19-year-olds who agree to participate in the randomly assigned interventions i.e. YouFB or adult FB |
| Implementation | Secondary outcomes | 2. Fidelity - whether all study and intervention components were implemented on schedule and as planned  3. Acceptability – the extent to which counsellors and beneficiaries perceive and value the intervention (YouFB).  4. Feasibility – the number of completed sessions. |
| Clinical | Primary outcome | - Proportion with SSQ-14 score ≥8 at endline. - Mean SSQ-14 score at endline (SD) |
| Clinical | Secondary outcomes | - Proportion with PHQ-9 score ≥11 at endline. - Mean PHQ-9 score at endline (SD) - Proportion with GAD-7 score ≥10 at endline. - Mean GAD-9 score at endline (SD) - Proportion with WHO-DAS score ≥20 at endline - Mean WHO-DAS score at endline (SD) |
| Cost |  | - Total program cost per year |

Supplementary Table 3: Allocation factors applied to each input type

| Assumption/Decision | Value |
| --- | --- |
| Discount rate | 3% |
| Exchange rate | $1 |
| Life of start-up costs (YouFB and FB) | 5 |
| Training economic life | 4 |
| Building economic life | 35 |
| Vehicle economic life | 10 |
| Furniture/equipment economic life | 5 |

The value of office equipment for non-programme office support staff was allocated proportionally based on staff level of effort. Predefined allocation factors including project M&E data, the proportion of LHW's trained, clients enrolled, and site distances from main office were used to allocate costs to their respective inputs (Supplementary Table 3). Training and programme supervision values depended on opportunity cost of staff and LHW time dedicated to these activities. Variation in costs across sites was assessed. All costs were analysed in 2020 United States dollars.

**Definitions of the cost categories and inputs**

**Start-up costs**

Start-up costs, including the costs incurred for sensitisation activities and initial training during the preparation period were treated as a capital cost as benefits of such investments would be expected to accrue to program over longer periods and therefore amortised at a 3% discount rate.

**Capital costs**

Capital costs include program building space and equipment. The benefits of such inputs also accrue to program over longer periods and were therefore amortised at a 3% discount rate.

**Recurrent costs**

Recurrent costs included personnel, stationery supplies, communication expenses, promotional materials such as hats, bags and t-shirts, vehicle and building operation and maintenance. Personnel costs include program management and staff time, psychologist time, supervision provided by Friendship Bench trainers, and other support and allowances for both youth and existing adult LHWs, clinicians and DHPOs.

Supplementary Table 4: Tidier Checklist for YouFB and Standard FB intervention

| **1. Brief name** | Youth Friendship Bench (YouFB) | Standard Friendship Bench |
| --- | --- | --- |
| **2. Why?** | The Youth Friendship Bench (YouFB) is an adaptation of the standard FB and was conceptualized to provide an alternative service for the youth. The intervention was developed using a theory of change workshop where young people and other stakeholders were engaged to provide input. The YouFB is a youth and community-based task-shifted intervention; it is delivered by youth peer counsellors. The YouFB aligns with a recommendation based on a case series on youth engagement to collaborate with young people early in the research process to allow for meaningful involvement. The YouFB was compared with the standard FB for 1) uptake, 2) reach, 3) effectiveness, and 4) economic costs. | The Friendship Bench (FB) was developed in Zimbabwe in 2006 to help bridge the treatment gap for CMDs and is highly effective in adults. It is an evidence-based, low-threshold psychological intervention. The FB is delivered to individuals and groups by community health workers (CHWs). It provides problem-solving therapy and behavioural activation within a community setting. Specifically, the Friendship Bench intervention focuses on exploring and understanding the clients' situational context through talk therapy, positive relational experience through being listened to, and intrapersonal growth towards strength and ability through goal-oriented learning. |
| **3. What materials are there?** | Youth FB training manual, FB card | Standard FB LHW training manual, FB card |
| **4. Procedures** | In the 13 communities offering the adapted YouFB intervention, services were provided by trained, mixed-sex YouFB "buddies". These were undergraduate psychology students serving a 10-month attachment with the Friendship Bench. They were selected following a competitive interview that assessed communication skills and a basic understanding of psychoeducation. The role of buddies was to raise mental health awareness within communities, screen young persons at risk of CMD, and provide FB sessions in clinical and non-clinical settings (e.g., churches, schools (after hours) and community centres). Young people at risk of CMD were enrolled into the YouFB intervention.  All 16–19-year-olds presenting to the buddies (intervention arm) were screened for CMD, and those at risk (scoring >8 on the SSQ) were invited to participate in the study. Buddies were supervised and supported by more experienced staff who were contacted by mobile phone if clients presented with "red flags" such as scores ≥11 on the SSQ-14, especially focusing on self-reports of suicidality or hallucinations. Those providing supervision included FB trainers and clinicians. | The Friendship Bench was conducted by existing adult female CHWs experienced in FB delivery in the 13 standard-of-care arm clinics. Adult CHWs received additional training around parental consent, reporting cases of abuse, confidentiality and dealing with minors in distress on providing the FB intervention to adolescents, ensuring they were aware of their specific needs.  All 16–19-year-olds presenting to the FB clinics (standard of care arm) were screened for CMD. Providers in the control arm were supervised and supported by more experienced staff who were contacted by mobile phone if clients presented with "red flags" such as scores ≥11 on the SSQ-14, especially focusing on self-reports of suicidality or hallucinations. Those providing supervision included adult CHWs who were not delivering the intervention in this trial and clinicians. |
| **5. Who provided?** | Youth Friendship Bench buddies (reimbursed $50/ month) | Adult community health workers (reimbursed $10/ month) |
| **6. How?** | Individual face-to-face sessions of 45-60mins | |
| **7. Where?** | 13 community clusters around Harare where the Friendship Bench is available were randomized to the YouFB intervention | 13 clinic clusters around Harare where the Friendship Bench is available were randomized to standard care |
| **8. When and how much?** | Buddies met with participants on a weekly basis for 6 face-to-face sessions over a 6-week period. | Adult CHWs met with participants on a weekly basis for 6 face-to-face sessions over a 6-week period. |
| **9. Tailoring** | The content of the intervention was tailored to suit young people. Participants with severe depression symptoms (score of 11 and above and or responding “Yes” to suicidal thoughts) were referred to the next level of care. | The content of the intervention was tailored for adults aged 18 and above. Participants with severe depression symptoms (score of 11 and above and or responding “Yes” to suicidal thoughts) were referred to the next level of care. |
| **10. Modifications** | No modifications were done on the intervention during the course of the study. | |
| **11. How well?: Planned** | To explore fidelity, we audio-recorded each participant's first counselling session with a YouFB buddy, and the assessment was done by FB trainers using a pre-designed checklist to establish fidelity. A process evaluation was also conducted to assess fidelity. | None. |
| **12. How well?: Actual** | The counselling sessions were delivered as planned. Additional training and support were provided to buddies to ensure intervention fidelity. | None. |
