# Supplementary material for: A cluster-randomized trial of interventions for adolescent mental disorders in Zimbabwe
Source: BMC Psychiatry. 2025 Jul 2;25:673. doi: 10.1186/s12888-025-06755-x (PMC12220635; doi:10.1186/s12888-025-06755-x)
Supplement: Supplementary file 2 — Supplementary Material 2 [file 12888_2025_6755_MOESM2_ESM.docx]

Supplementary File S2: Quantitative data collection tool

New Project 2018

Research assistant

Please enter PTID

*Format: FBZ-nnnn*

Clinic Name


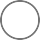
 Avondale PCC
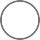
 Mt Pleasant


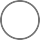
 Borrowdale FHS
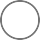
 Hatfield PCC
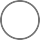
 Belvedere


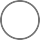
 Arcadia


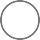
 Mabelreign Satelite
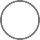
 Sunningdale


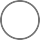
 Malborough Satelite
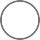
 Waterfalls


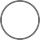
 Dzivarasekwa Poly
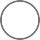
 Glenview Poly


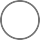
 Kuwadzana Poly
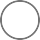
 Highfields Poly
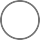
 Warren Park Poly
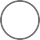
 Rutsanana Poly
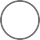
 Glen View Satelite
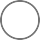
 Budiriro Poly


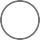
 Mabvuku Poly


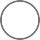
 Glennorah Satelite
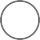
 Mabvuku Satelite
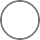
 Kambuzuma Poly
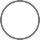
 Western Triangle
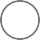
 Mufakose Poly


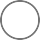
 Mbare Polyclinic (Edith Opperman)
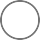
 Hatcliffe Polyclinic


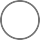
 St Marys
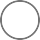
 Zengeza 3
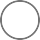
 Seke North
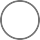
 Seke South

SOCIODEMOGRAPHIC DATA

1. Gender


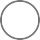
 Male
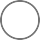
 Female

1. What is your age?


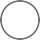
 16


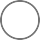
 17


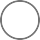
 18


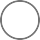
 19

1. How many people are currently living in your household, including yourself?


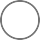
 1-2


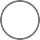
 3-4


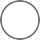
 5-8


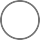
 9+

1. What is the highest level of education you have completed?


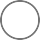
 Below Grade 7


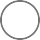
 Grade 7
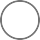
 O'Level
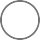
 A'Level


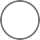
 Tertiary Level

1. What is your current marital status?


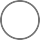
 Single
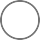
 Married
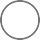
 Divorced
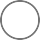
 Widowed

INSTRUCTION: This is the time to handover the tablet to the participant to continue with the interview

1. Are you currently employed?


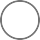

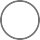
 No Yes

6a. Indicate your Monthly Income bracket?


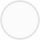
 $1-$50
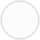
 $51-$100
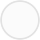
 $101-$200
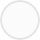
 $201-$499
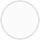
 $500+

1. Do you smoke?


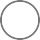
 No
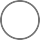
 Yes

1. Do you take alcohol?


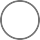
 No
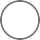
 Yes

8a. How often


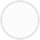
 Daily
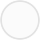
 Weekly
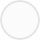
 Monthly

1. Do you take drugs/substances?


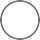
 No
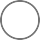
 Yes

9a. what do you take?


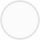
 Marijuana
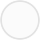
 Broncho


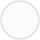
 Prescription Drugs
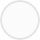
 Cocaine


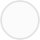
 Cocktail of Drugs
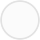
 Other

1. What led you to take these drugs, substances and alcohol?


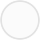
 Peer pressure
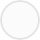
 Willingly


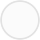
 Stress

1. Does anyone from your family have a history of taking substances, alcohol or smoking?


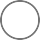

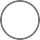
 No Yes

1. How many children do you have?


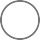
 0


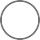
 1


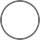
 2


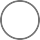
 3


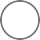
 4+

1. Do you know your HIV Status


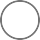
 No
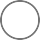
 Yes

13a. Are you free to disclose your status?


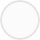
 No
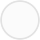
 Yes

13b. What is your HIV status?


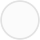
 HIV negative
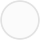
 HIV positive

13c. Are you on ART?


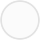
 No
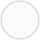
 Yes

1. What is your Religion?


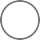
 Catholic
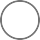
 Muslim
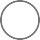
 Adventist
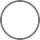
 Methodist


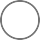
 Pentecostal
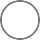
 None


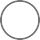
 Other

14a. Other specify

The next statements ask you to think about how you felt in the LAST WEEK. For each statement, Please press on one box only.

SSQa. In the last week, there were times in which I was thinking deeply or thinking about many things.


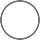

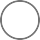
 No Yes

SSQb. In the last week, I found myself sometimes failing to concentrate


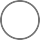
 No
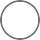
 Yes

SSQc. In the last week, I lost my temper or got annoyed over trivial matters

No Yes

SSQd. In the last week, I had nightmares or bad dreams

No Yes

SSQe. In the last week, I sometimes saw or heard things which others could not see or hear

No Yes

SSQf. In the last week, my stomach was aching

No Yes

SSQg. In the last week, I was frightened by trivial things

No Yes

SSQh. In the last week, I sometimes failed to sleep or lost sleep

No Yes

SSQi. In the last week, there were moments when I felt life was so tough that I cried or wanted to cry

No Yes

SSQj. In the last week, I felt run down (tired)

No Yes

SSQk. In the last week, There were times I felt like committing suicide

No Yes

SSQl. In the last week, I was generally unhappy with things that I would be doing each day

No Yes

SSQm. In the last week, My work was lagging behind

No Yes

SSQn. In the last week, I felt I had problems in deciding what to do

No Yes

Over the last two weeks, how often have you been affected by any of the following problems?

GAD01. Feeling anxious, nervous or on edge?

Not at all sure Several days

Over half the days Nearly every day

GAD02. Not being able to stop or control worrying?

Not at all sure Several days

Over half the days Nearly every day

GAD03. Worrying too much about different things?

Not at all sure Several days

Over half the days Nearly every day

GAD04. Trouble relaxing?

Not at all sure Several days

Over half the days Nearly every day

GAD05. Being so restless that it is hard to sit still?

Not at all sure Several days

Over half the days Nearly every day

GAD06. Becoming easily annoyed or irritable?

Not at all sure Several days

Over half the days Nearly every day

GAD07. Feeling afraid as if something awful might happen?

Not at all sure Several days

Over half the days Nearly every day

GAD08. If you checked off any problems, how difficult have these made it for you to do your work, take care of things at home or get along with other people?

Not at all Several days

More than half the days Nearly every day

PHQ9. Over the LAST 2 WEEKS, how often have you been affected by any of the following problems?

PHQ9a. Little interest or pleasure in doing things.

Not at all Several days

More than half the days Nearly every day

PHQ9b. Feeling down, depressed, or hopeless.

Not at all Several days

More than half the days Nearly every day

PHQ9c. Trouble falling or staying asleep, or sleeping too much.

Not at all Several days

More than half the days Nearly every day

PHQ9d. Feeling tired or having little energy.

Not at all Several days

More than half the days Nearly every day

PHQ9e. Poor appetite or overeating.

Not at all Several days

More than half the days Nearly every day

PHQ9f. Feeling bad about yourself — or that you are a failure or have let yourself or your family down.

Not at all Several days

More than half the days Nearly every day

PHQ9g. Trouble concentrating on things, such as reading the newspaper or watching television.

Not at all Several days

More than half the days Nearly every day

PHQ9h. Moving or speaking so slowly that other people could have noticed? Or the opposite — being so fidgety or restless that you have been moving around a lot more than usual.

Not at all Several days

More than half the days Nearly every day

PHQ9i. Thoughts that you would be better off dead or of hurting yourself in some way.

Not at all Several days

More than half the days Nearly every day

This questionnaire asks about difficulties due to health conditions. Health conditions include diseases or illnesses, other health problems that may be short or long lasting, injuries, mental or emotional problems, and problems with alcohol or drugs.

D1_1. In the past month how much difficult did you have in concentrating on doing something for ten minutes?

None Mild

Moderate Severe

Extreme or cannot do

D1_2. In the past month how much difficult did you have in remembering to do important things?

None Mild

Moderate Severe

Extreme or cannot do

D1_3. In the past month how much difficult did you have in analysing and finding solutions to problems in day-to-day life?

None Mild

Moderate Severe

Extreme or cannot do

D1_4. In the past month how much difficult did you have in learning a new task, for example, learning how to get to a new place?

None Mild

Moderate Severe

Extreme or cannot do

D1_5. In the past month how much difficult did you have in generally understanding what people say?

None Mild

Moderate Severe

Extreme or cannot do

D1_6. In the past month how much difficult did you have in starting and maintaining a conversation?

None Mild

Moderate Severe

Extreme or cannot do

D2_1. In the past month how much difficult did you have in standing for long periods such as 30 minutes?

None Mild

Moderate Severe

Extreme or cannot do

D2_2. In the past month how much difficult did you have in standing up from sitting down?

None Mild

Moderate Severe

Extreme or cannot do

D2_3. In the past month how much difficult did you have in moving around inside your home?

None Mild

Moderate Severe

Extreme or cannot do

D2_4. In the past month how much difficult did you have in getting out of your home?

None Mild

Moderate Severe

Extreme or cannot do

D2_5. In the past month how much difficult did you have in walking a long distance such as a kilometre (or equivalent)?

None Mild

Moderate Severe

Extreme or cannot do Self-care

D3_1. In the past month how much difficult did you have in washing your whole body?

None Mild

Moderate Severe

Extreme or cannot do

D3_2. In the past month how much difficult did you have in getting dressed?

None Mild

Moderate Severe

Extreme or cannot do

D3_3. In the past month how much difficult did you have in eating?

None Mild

Moderate Severe

Extreme or cannot do

D3_4. In the past month how much difficult did you have in staying by yourself for a few days?

None Mild

Moderate Severe

Extreme or cannot do

D4_1. In the past month how much difficult did you have in dealing with people you do not know?

None Mild

Moderate Severe

Extreme or cannot do

D4_2. In the past month how much difficult did you have in maintaining a friendship?

None Mild

Moderate Severe

Extreme or cannot do

D4_3. In the past month how much difficult did you have in getting along with people who are close to you?

None Mild

Moderate Severe

Extreme or cannot do

D4_4. In the past month how much difficult did you have in making new friends?

None Mild

Moderate Severe

Extreme or cannot do

D4_5. In the past month how much difficult did you have in sexual activities?

None Mild

Moderate Severe

Extreme or cannot do

D5_1. In the past month how much difficult did you have in taking care of your household responsibilities?

None Mild

Moderate Severe

Extreme or cannot do

D5_2. In the past month how much difficult did you have in doing most important household tasks well?

None Mild

Moderate Severe

Extreme or cannot do

D5_3. In the past month how much difficult did you have in getting all the household work done that you needed to do?

None Mild

Moderate Severe

Extreme or cannot do

D5_4. In the past month how much difficult did you have in getting your household work done as quickly as needed?

None Mild

Moderate Severe

Extreme or cannot do

D5_5. In the past month how much difficult did you have in your day-to-day work/school?

None Mild

Moderate Severe

Extreme or cannot do

D5_6. In the past month how much difficult did you have in doing your most important work/school tasks well?

None Mild

Moderate Severe

Extreme or cannot do

D5_7. In the past month how much difficult did you have in getting all the work done that you need to do?

None Mild

Moderate Severe

Extreme or cannot do

D5_8. In the past month how much difficult did you have in getting your work done as quickly as needed?

None Mild

Moderate Severe

Extreme or cannot do

D6_1. How much of a problem did you have in joining in community activities (for example, festivities, religious or other activities) in the same way as anyone else can?

None Mild

Moderate Severe

Extreme or cannot do

D6_2. How much of a problem did you have because of barriers or hindrances in the world around you?

None Mild

Moderate Severe

Extreme or cannot do

D6_3. How much of a problem did you have living with dignity because of the attitudes and actions of others?

None Mild

Moderate Severe

Extreme or cannot do

D6_4. How much time did you spend on your health condition, or its consequences?

None Mild

Moderate Severe

Extreme or cannot do

D6_5. How much have you been emotionally affected by your health condition?

None Mild

Moderate Severe

Extreme or cannot do

D6_6. How much has your health been a drain on the financial resources of you or your family?

None Mild

Moderate Severe

Extreme or cannot do

D6_7. How much of a problem did your family have because of your health problems?

None Mild

Moderate Severe

Extreme or cannot do

D6_8. How much of a problem did you have in doing things by yourself for relaxation or pleasure?

None Mild

Moderate Severe

Extreme or cannot do

H1. Overall, in the past 30 days, how many days were these difficulties present

H2. In the past 30 days, for how many days were you totally unable to carry out your usual activities or work because of any health condition?

H3. In the past 30 days, not counting the days that you were totally unable, for how many days did you cut back or reduce your usual activities or work because of any health condition?

Thank you. This the end . Call Frienship Bench Worker to help you

Total SSQ Score NaN

SSQ Score on Q5 is

SSQ Score on Q11 is
