# Supplementary material for: A cluster-randomized trial of interventions for adolescent mental disorders in Zimbabwe
Source: BMC Psychiatry. 2025 Jul 2;25:673. doi: 10.1186/s12888-025-06755-x (PMC12220635; doi:10.1186/s12888-025-06755-x)
Supplement: Supplementary file 3 — Supplementary Material 3 [file 12888_2025_6755_MOESM3_ESM.docx]

Supplementary File S3: Qualitative Interview guides

**IN-DEPTH INTERVIEW GUIDE: LAY HEALTH WORKERS (LHW)**

*(****Note to interviewer:*** *Questions in bullet form are meant to be probes. They do not have to be asked as they appear here. Rather, phrase/order questions according to flow of discussion).*

1. *Main question*: Can you tell me about the FB program for adolescents you have been implementing?

- What works? What does not work? *Why?*

1. *Main* question: How easy or difficult has it been to implement the approach as you were trained or as expected? *Why?*

- What are the things you do as per your training/superior's expectations?
- What are the things you do differently? Why?
- What are the challenges you encounter? How do you deal with these challenges?

1. *Main* question: What do you think about the acceptability of the program?

- To what extent is it acceptable to various subpopulations of adolescents?
- We have seen that a high number of potential participants might be unwilling to participate in the study. Why is this?
- What would make them more willing to participate?

1. Main question: To what extent does acceptability vary by cadre (i.e. YLHW vs. LHW)? *Please explain.*
2. *Main* question: It appears more male than female adolescents are being recruited? *Why is this?*

- What could be done to sustain uptake among males?
- What could be done to enhance uptake among females?

1. *Main* question: To what extent are participants likely to continue with the program once they have enrolled?

- Why do some keep going?

1. *Main* question: We have seen that some participants who have scheduled sessions do not come back. *Why is this?*

- Why could be done to ensure they continue with sessions?

1. *Main question*: In your view, what has been the impact of the program on those providing it?

- What has been the impact on recipients?
- What could be done for it to be more impactful (to clients)?

1. *Main question*: What are the limitations of the community component?

- Under what circumstances do adolescents end up seeking help at the clinic (because their problems cannot be addressed by the community component)?

1. *Main question*: To what extent could the program be implemented in a different setting?

- If implemented in a different setting, what needs to be kept?
- If implemented in a different setting, what needs to change?

1. *Main question*: We have seen that program reach is sometimes overstated. Why would this happen?
2. *Main question*: Is there anything else you want to tell me about the FB program for adolescents?

*(****Note to interviewer:*** *Questions in bullet form are meant to be probes. They do not have to be asked as they appear here. Rather, phrase/order questions according to flow of discussion).*

**INTERVIEW GUIDE: YOUTH LAY HEALTH WORKERS (BUDDIES)**

*(****Note to interviewer:*** *Questions in bullet form are meant to be probes. They do not have to be asked as they appear here. Rather, phrase/order questions according to flow of discussion).*

1. *Main question*: Can you tell me about the FB program for adolescents you have been part of?

- What did you like about it? What didn't you like?

1. *Main question*: If you were telling your friend about the program, what would you say to them?

- ***Let's imagine I am your friend, now tell me about the FB program for adolescents (good and not so good) - let them role play***

1. In your view, what has been the impact of the program on those receiving it?

- What has been the impact to your colleagues?
- What has been the impact to you?

3. *Main question*: Which aspects of the program are most impactful or helpful? *Why?*

- Which ones are least helpful? *Why?*

1. *Main* question: What do you think about the fact that the program was implemented by young people?

- What was good about it? What was not good about it?
- What are some of the things you wish they could do (i.e. that they didn't do)?
- What are some of the things you wish they could do better?

1. Main question: If the program was offered by adult LHWs, how acceptable would it be to adolescents?

- What are some of the things they would not tell the adult LHWs?
- What are some of the things adult LHWs would be better at (compared to YLHWs)?

1. *Main* question: It appears more male than female adolescents are being recruited? *Why is this?*

- What could be done to improve uptake among females?

1. *Main* question: To what extent are participants likely to continue with the program once they have enrolled?

- Why do some keep going?

1. *Main* question: We have seen that some participants who have scheduled sessions do not come back. *Why is this?*

- What could be done to ensure they continue with sessions?

1. *Main question*: We have seen that some participants remain with challenges even after receiving the program. Why is that?

- What could be done to help these participants?

1. *Main question*: Is there anything else you want to tell me about the FB program for adolescents?
